# Supplementary material for: Age acquired skewed X chromosome inactivation is associated with adverse health outcomes in humans
Source: eLife. 2022 Nov 22;11:e78263. doi: 10.7554/eLife.78263 (PMC9681199; doi:10.7554/eLife.78263)
Supplement: Supplementary file 1. [file elife-78263-supp1.docx]

Supplementary Data – *Roberts et al*

**Tables**

**Table S1**: Numbers of cancer diagnoses recorded in 10-year follow-up by organ/site. These numbers represent the first cancer diagnosis for each individual.

|  | Number of cancer diagnoses | | |
| --- | --- | --- | --- |
| Organ/Tissue | Total | In individuals with XCI-skew | In individuals with random XCI |
| Bowel | 11 | 6 | 5 |
| Breast | 26 | 14 | 12 |
| Endocrine | 1 | 1 | 0 |
| Female Reproductive Organs | 4 | 2 | 2 |
| Haematopoietic / Lymphoid Tissues | 4 | 2 | 2 |
| Oesophagus | 1 | 0 | 1 |
| Oral Cavity | 1 | 1 | 0 |
| Ovary | 4 | 1 | 3 |
| Pancreas | 1 | 0 | 1 |
| Secondary/ill-defined/Unspecified site | 1 | 1 | 0 |
| Skin melanoma | 3 | 1 | 2 |
| Urinary Tract | 1 | 1 | 0 |
| **Total** | **58** | **30** | **28** |
